# Supplementary material for: UBE2G1 governs the destruction of cereblon neomorphic substrates
Source: eLife. 2018 Sep 20;7:e40958. doi: 10.7554/eLife.40958 (PMC6185104; doi:10.7554/eLife.40958)
Supplement: Peer review history

# Decision letter

**DOI:** 10.7554/eLife.40958.032

In the interests of transparency, eLife includes the editorial decision letter and accompanying author responses. A lightly edited version of the letter sent to the authors after peer review is shown, indicating the most substantive concerns; minor comments are not usually included.

Thank you for submitting your article "UBE2G1 Governs the Destruction of Cereblon Neomorphic Substrates" for consideration by *eLife*. Your article has been reviewed by three peer reviewers, including Wade Harper as the Reviewing Editor and Reviewer #1, and the evaluation has been overseen by Philip Cole as the Senior Editor.

The reviewers have discussed the reviews with one another and the Reviewing Editor has drafted this decision to help you prepare a revised submission.

Summary:

In this manuscript, Lu et al. reported the identification of two ubiquitin-conjugating E2 enzymes, UBE2G1 and UBE2D3, that function cooperatively with the CRL4-DDB1-CRBN ubiquitin E3 ligase complex to polyubiquitinate neomorphic substrates in the presence of immunomodulatory drugs (IMiDs) and their derivative compounds. The authors identified the two E2s with a CRISPR-CAS screen, in which the effect on the pomalidomide-induced IKZF1 degradation was monitored with all human E2 enzymes individually knocked out. With both in vitro and in vivo polyubiquitination assays, the authors have further revealed that the two E2s, UBE2D3 and UBE2G1, act sequentially to build mono- and K48-linked polyubiquitin chain on the CRBN substrates. Last but not least, the authors provided evidence suggesting that UBE2G1 downregulation confers drug resistance to lenalidomide and pomalidomide, but not CC-220, a more potent IMiD derivative.

Overall speaking, the significance of the study is three-fold. First, proper mapping of cognate E2s for specific E3s has been a major gap in the field of protein ubiquitination. Although several E2s, such as CDC34 and RAD6, have been successfully paired with their E3s, thanks to early genetic studies, the majority of E2s and E3s have not been functionally associated. This problem compromises the significance of many biochemical studies, in which the activity of an E3 is usually established or examined with a promiscuous, but not necessarily relevant, E2. In the case of CRL E3s, which share the same RBX1 RING subunit, it has been particularly perplexing that CDC34 works well with SCF/CRL1, but not other CRL subfamilies. This study has elegantly addressed this question, at least for CRL4. Second, the sequential actions of UBE2D3 and UBE2G1 provide an interesting explanation for why the CRL4-CRBN E3 requires two different E2 enzymes to polyubiquitinate its substrates. The mechanism is highly reminiscent of the sequential functions of UBE2D3 and CDC34 with SCF. Third, the CRL4-DDB1-CRBN E3 complex represents the first ubiquitin ligase which is reprogrammed by a class of clinically used drugs for neo-substrate degradation. The findings of this study offer us a better understanding of drug resistance mechanisms and might guide the development of future generations of IMiDs.

Essential revisions:

After reviewer consultation, it is recommended that the paper be accepted with only minor revisions (see below), which hopefully will allow rapid publication. Although a recent previous paper reported similar results, the current study was clearly performed contemporaneously, is highly complementary, and fits easily within *eLife*'s editorial policy on overlapping papers.

1) The Petroski and Deshaies review used for CRLs is terribly out of date, being published in 2005. There are several more recent CRL reviews that could be used instead or in addition to this paper.

2) In the last paragraph of the Introduction, the authors discuss the sequential reaction of UbcH5 and Cdc34 in the context of specific SCFs. This however may be a minor pathway compared with the apparently much more widespread use of the ARIH1 co-E3 for priming substrate ubiquitylation (see Pubmed: 27565346). Minimally the ARIH1 model should be mentioned as an additional mechanism for initial transfer of Ub to substrate followed by chain assembly with Cdc34. In reality, since ARIH1 is a RBR E3 (receiving Ub from UbcH7), once could consider this mechanism as just being 2 different E2s.

3) In Figure 3, the cell lines should be indicated with the gels, not just in the legend in order to make it easier to follow.

4) In the last paragraph of the subsection “UBE2G1 is the dominant ubiquitin E2 enzyme that governs the destruction of cereblon neomorphic substrates induced by cereblon modulating agents”, the authors conclude that UBE3G1 might be specific for CUL4 E3s given that its deletion didn't affect 2 SCF targets. However, there are no experiments that rule out a role for other CRLs, so this sentence should be modified. It is also not clear whether UBE2G1 works for some or all other CRL4 complexes normally.

5) Abstract: The authors claim that their data suggest that "loss of UBE2G1 activity might be a resistance mechanism to drugs that hijack the CRL4CRBN to eliminate disease-driving proteins, and that this resistance mechanism can be overcome by next-generation CMs that destroy the same targeted protein more effectively". The statement should be removed from the Abstract as there is no data showing that a change in UBE2G1 activity actually occurs during the development of drug resistance.

6) In the second paragraph of the subsection “UBE2G1 is the dominant ubiquitin E2 enzyme that governs the destruction of cereblon neomorphic substrates induced by cereblon modulating agents”, the use of U937 cells in the CRISPR screen needs to be justified.

7) Figure 1—figure supplement 2 was mentioned before Figure 1—figure supplement 1C. Additionally, in the subsection “UBE2G1 is the dominant ubiquitin E2 enzyme that governs the destruction of cereblon neomorphic substrates induced by cereblon modulating agents”, 'co-treatment' is confusing. Is there another drug in the experiment?

8) Subsection “UBE2G1 is the dominant ubiquitin E2 enzyme that governs the destruction of cereblon neomorphic substrates induced by cereblon modulating agents”, fourth paragraph. Although the authors' results clearly suggest that there should be at least another E2 that act in parallel to UBE2G1 to degrade IKZF1 (the effect of UbE2G1 knockout is much smaller than that of E3 knockout), surprisingly, the double sgRNA screen did not reveal any additional E2s that can mediate IKZF1 degradation. It is also surprising that knockout of Ube2D3 could enhance the effect of UBE2G1 knockout although the model suggests that the two enzymes work in sequence. I feel that these data are not explained well. The authors should keep in mind that UBE2D3 is a quite promiscuous E2 and works with many E3s. Thus, the effect of UBE2D3 knockout may not always be direct. This could be addressed to a large degree by re-wording of the text.

9) Subsection “UBE2G1 is the dominant ubiquitin E2 enzyme that governs the destruction of cereblon neomorphic substrates induced by cereblon modulating agents”, fifth paragraph. Please link each substrate with a specific panel. It is hard to scan through panels scattered in 4 different figures in order to follow each of the substrates mentioned here.

10) The authors refer to different lanes in immunoblots, but the lanes are not labeled.

11) Subsection “UBE2G1 mediates the ubiquitination of cereblon neomorphic substrates”, last paragraph: why does expression of UBE2D3 in UBE2G1 knockout cells partially rescue the degradation phenotype?

# Reply: Author response

**DOI:** 10.7554/eLife.40958.033

1) The Petroski and Deshaies review used for CRLs is terribly out of date, being published in 2005. There are several more recent CRL reviews that could be used instead or in addition to this paper.

Thanks for pointing out this obvious negligence to us. A more recent CRL review article (Lehti et al., 2013) is now cited as well.

2) In the last paragraph of the Introduction, the authors discuss the sequential reaction of UbcH5 and Cdc34 in the context of specific SCFs. This however may be a minor pathway compared with the apparently much more widespread use of the ARIH1 co-E3 for priming substrate ubiquitylation (see Pubmed: 27565346). Minimally the ARIH1 model should be mentioned as an additional mechanism for initial transfer of Ub to substrate followed by chain assembly with Cdc34. In reality, since ARIH1 is a RBR E3 (receiving Ub from UbcH7), once could consider this mechanism as just being 2 different E2s.

Thanks for the great suggestion. The sequential ubiquitination of SCF substrates carried out by ARIH1/UbcH7 and CRL/Cdc34 is now discussed in the Introduction.

3) In Figure 3, the cell lines should be indicated with the gels, not just in the legend in order to make it easier to follow.

We apologize for the confusion. All gels are now labeled with the cell line names indicated at the top of each figure panel.

4) In the last paragraph of the subsection “UBE2G1 is the dominant ubiquitin E2 enzyme that governs the destruction of cereblon neomorphic substrates induced by cereblon modulating agents”, the authors conclude that UBE3G1 might be specific for CUL4 E3s given that its deletion didn't affect 2 SCF targets. However, there are no experiments that rule out a role for other CRLs, so this sentence should be modified. It is also not clear whether UBE2G1 works for some or all other CRL4 complexes normally.

We agree with the reviewers’ comment. We have changed the wording to “[…] indicating the specific regulation of CRL4^CRBN^ by UBE2G1”.

5) Abstract: The authors claim that their data suggest that "loss of UBE2G1 activity might be a resistance mechanism to drugs that hijack the CRL4CRBN to eliminate disease-driving proteins, and that this resistance mechanism can be overcome by next-generation CMs that destroy the same targeted protein more effectively". The statement should be removed from the Abstract as there is no data showing that a change in UBE2G1 activity actually occurs during the development of drug resistance.

We agree with the reviewers that it is merely a speculation that UBE2G1 loss could contribute to resistance to cereblon-repurposing drugs in human patients, even though loss of UBE2G1 expression in human cancer cell lines tested did confer resistance to immunomodulatory drugs and their derivatives. We have removed the claim and changed the wording to “Collectively, it will be of fundamental interest to explore if loss of UBE2G1 activity is linked to clinical resistance to drugs that hijack the CRL4^CRBN^ to eliminate disease-driving proteins.”

6) In the second paragraph of the subsection “UBE2G1 is the dominant ubiquitin E2 enzyme that governs the destruction of cereblon neomorphic substrates induced by cereblon modulating agents”, the use of U937 cells in the CRISPR screen needs to be justified.

The cell line selection criteria we applied for the CRISPR screen were the following:

1) A cell line that expresses endogenous IKZF1, which can be used as an internal control to determine the expression level of the ePL-tagged IKZF1 stability reporter. We tried to keep the expression level of the ePL-IKZF1 fusion protein comparable to that of endogenous IKZF1, so that the ePL-IKZF1 fusion protein will not saturate the ubiquitination/degradation machinery controlling the pomalidomide-induced IKZF1 degradation. Thus, the ePL-IKZF1 reporter could be used to monitor the degradation of IKZF1 under near-physiological conditions. For this reason, myeloma, DLBCL and AML cell lines which express endogenous IKZF1 are our best screen models. We successfully generated a U937 cell line stably expressing ePL-IKZF1 at a level below its endogenous protein (Figure 1—figure supplement 1B).

2) A cell line in which pomalidomide could induce robust degradation of IKZF1 without affecting cell proliferation and survival during the course of the ePL assay. Since a cell-autonomous effect of pomalidomide on cell proliferation and/or survival would obscure the readout of the ePL-IKZF1 reporter, myeloma and DLBCL cell lines which depend on IKZF1 and IKZF3 for survival were not chosen for the screen. It was shown previously that lenalidomide could induce the degradation of IKZF1 in a neddylation- and proteasome-dependent manner in U937 cells (Lu et al., Science 2014). We have confirmed that pomalidomide could also induce the cereblon-dependent degradation of IKZF1 (Figure 1—figure supplement 1) without affecting cellular fitness (data not shown).

3) A cell line that has a high lentiviral transduction efficiency and quickly recovers after lentiviral infection. Lentiviral transduction inhibits cell proliferation for several days in many DLBCL and myeloma cell lines. Moreover, considering the potential essential function of many E2s for cell proliferation and/or survival, we think that long term culture of cells following CRISPR knockout of essential E2s will lead to enrichment of clones with less inactivation of the respective E2s, and therefore we could potentially underestimate their effects on pomalidomide-induced IKZF1 degradation. In our hand, U937 AML cell line fits this criteria better than other AML cell lines tested.

Justification for the use of U937 cells is now added in the main text: “We chose U937 cells for assessing the effect of individual gene knockout on POM-induced IKZF1 degradation, because we were able to express the ePL-tagged IKZF1 fusion protein below the level of endogenous IKZF1 protein, and POM can induce the efficient degradation of both endogenous and ePL-tagged IKZF1 (Figure 1—figure supplement 1).”

7) Figure 1—figure supplement 2 was mentioned before Figure 1—figure supplement 1C. Additionally, in subsection “UBE2G1 is the dominant ubiquitin E2 enzyme that governs the destruction of cereblon neomorphic substrates induced by cereblon modulating agents”, 'co-treatment' is confusing. Is there another drug in the experiment?

Figure 1—figure supplement 1C is now changed to Figure 1—figure supplement 2V.

We apologize for the confusion. “Co-treatment” means that U937 cells were incubated with both pomalidomide and MLN4924. The text now reads:

“Indeed, MLN4924, an inhibitor of NEDD8-activating enzyme (Soucy et al., 2009), prevented the degradation of ePL-IKZF1 induced by POM (Figure 1—figure supplement 2V).”

8) Subsection “UBE2G1 is the dominant ubiquitin E2 enzyme that governs the destruction of cereblon neomorphic substrates induced by cereblon modulating agents”, fourth paragraph. Although the authors' results clearly suggest that there should be at least another E2 that act in parallel to UBE2G1 to degrade IKZF1 (the effect of UbE2G1 knockout is much smaller than that of E3 knockout), surprisingly, the double sgRNA screen did not reveal any additional E2s that can mediate IKZF1 degradation. It is also surprising that knockout of Ube2D3 could enhance the effect of UBE2G1 knockout although the model suggests that the two enzymes work in sequence. I feel that these data are not explained well. The authors should keep in mind that UBE2D3 is a quite promiscuous E2 and works with many E3s. Thus, the effect of UBE2D3 knockout may not always be direct. This could be addressed to a large degree by re-wording of the text.

We agree with reviewers’ comment that our observation is quite perplexing. In agreement with the independent finding made by Dr. Ben Ebert’s lab (Sievers QL et al., Blood 2018), UBE2G1 could promote the K48-linked ubiquitin chain assembly on IKZF1 only when primed with monoubiquitin(s) by UBE2D3. Logically one would expect knockout of UBE2D3 should have similar effect on the degradation of IKZF1 as did UBE2G1 inactivation. We also agree with the reviewers that one should expect to identify additional E2s mediating IKZF1 ubiquitination in addition to UBE2D3, based on the differential effect of UBE2G1 knockout and CRBN knockout.

One explanation, as the reviewer clearly pointed out, is that there might be another E2 that we missed in the screen, and the effect of UBE2D3 knockout could be indirect. Given the redundant role of UBE2D family proteins in catalyzing the in vitro ubiquitination of GSPT1 (Figure 4—figure supplement 2), and the potential redundancy between UBE2G1 and its paralog UBE2G2 (although this mechanism has not been validated in our manuscript), we proposed several hypotheses to address the reviewers’ concern (Discussion section).

9) Subsection “UBE2G1 is the dominant ubiquitin E2 enzyme that governs the destruction of cereblon neomorphic substrates induced by cereblon modulating agents”, fifth paragraph. Please link each substrate with a specific panel. It is hard to scan through panels scattered in 4 different figures in order to follow each of the substrates mentioned here.

Thanks for the great suggestion. Each substrate is now linked to specific panels. The text now reads:

“Ablation of UBE2G1 significantly diminished the degradation of IKFZ1 (Figures 3A, and Figure 3—figure supplement 1A, B), IKZF3 (Figures 3A, and Figure 3—figure supplement 1A, B) and ZFP91 (Figures 7B, and Figure 7—figure supplement 1B, D) by LEN, POM, and CC-220, as well as CK1α degradation (Figures 7B, and Figure 7—figure supplement 1B, D) by LEN in OPM2, DF15 and MM1S myeloma cells.”

10) The authors refer to different lanes in immunoblots, but the lanes are not labeled.

We apologize for our negligence. The lanes in Figures 4A-E, Figure 4—figure supplement 1C, 2B, Figures 5A-C, and Figure 5—figure supplement 1A-C are now labeled.

11) Subsection “UBE2G1 mediates the ubiquitination of cereblon neomorphic substrates”, last paragraph: why does expression of UBE2D3 in UBE2G1 knockout cells partially rescue the degradation phenotype?

Although UBE2D3 alone could only promote the monoubiquitination of CRBN neosubstrates IKZF1 and GSPT1 in vitro (Figure 4A and 4B), overexpressed UBE2D3 induced monoubiquitination and to a lesser extent polyubiquitination of IKZF1 in 293T UBE2G1-/- cells (Figure 5B). It is possible that other E2s such as UBE2G2 could still cooperate with overexpressed UBE2D3 to enhance the rate of CRBN neosubstrate ubiquitination degradation. Alternatively, UBE2D3 itself could indirectly promote neosubstrate ubiquitination and degradation in cells, though this effect may not be physiologically relevant. Nevertheless, the differential effect of FLAG-tagged UBE2D3 wild-type and C85S mutant on GSPT1 degradation in 293T UBE2G1 -/- cells (Figure 5—figure supplement 1B), suggests that FLAG-UBE2D3 is a functional enzyme.

According to the *eLife*’s publication guideline, we made a few additional changes: 1) rename all supplementary figures and supplementary file; 2) add raw source data for all graphs; 3) reword the Abstract due to word count limit; 4) add a key resources table.
